# Editorial: Integrating multimodal approaches to unravel neural mechanisms of learning and cognition

**DOI:** 10.3389/fneur.2026.1753883

**Published:** 2026-01-26

**Authors:** Tao Xu, Fang Luo, Ying Cui, Yun Zhou

**Affiliations:** 1 School of Software, Northwestern Polytechnical University, Xi'an, Shaanxi, China; 2 Faculty of Psychology, Beijing Normal University, Beijing, China; 3 Educational Psychology Department, Faculty of Education, University of Alberta, Edmonton, AB, Canada; 4 Faculty of Education, Shaanxi Normal University, Xi'an, Shaanxi, China

**Keywords:** assessment of learning and cognitive states, multi-modal approaches, brain–computer interfaces, eye-tracking, neuroimaging, large language model

## 1 Introduction

Understanding how learning and cognition unfold in real time has long been a central aim of cognitive neuroscience (1). Considerable progress has been achieved in elucidating core cognitive functions such as attentional control (2, 3), working memory (4), and executive functioning (5, 6). However, the neural mechanisms that integrate these processes as learners perceive, consolidate, and apply information in authentic contexts remain only partially understood. Recent work has begun to trace these dynamics by employing multimodal approaches that combine diverse signals, including neural activity, eye movements, facial expressions, and interactive behaviors, to capture the complexity of learning in action (7–9).

Advancing this agenda requires methodological frameworks that can capture learning and cognitive states as multimodal and dynamically evolving phenomena. This necessitates the integration of high-temporal-resolution techniques (e.g., EEG, eye-tracking) with hemodynamic imaging methods (e.g., fNIRS, MRI) and computational analyses of behavior, enabling the derivation of quantitative indicators that are reliable across tasks, contexts, and populations. The convergence of these state-of-the-art technologies now makes such integration increasingly feasible. EEG and eye-tracking can capture rapid fluctuations (10, 11); fNIRS and MRI can identify network-level activations (12, 13); and large language models can process and interpret rich behavioral data (14, 15), from spoken explanations to written reflections, at scale. When synchronized along common temporal axes and analytic pipelines, these multimodal data streams can uncover coherent neural-behavioral signatures spanning information acquisition, consolidation, and application, thereby enabling real-time measurement, adaptive feedback, and the development of neuroscience-informed educational interventions.

This editorial introduces the Research Topic “Investigating Learning and Cognitive States Using Multimodal Approaches,” which seeks empirical studies, methodological innovations, and reviews that: (a) apply multimodal approaches to recognize and assess learning and cognitive states; (b) examine the use and implications of EEG, fNIRS, MRI, eye-tracking, and LLMs in learning contexts; (c) develop quantitative metrics and validation strategies for cognitive state measurement; (d) present novel technologies for studying learning and cognition; and (e) leverage neuroimaging for assessing learning-relevant states and outcomes. Contributions that provide open resources, reproducible workflows, and translational pathways to educational practice are especially encouraged.

## 2 Contributions of the papers to this Research Topic

The 10 papers selected for this Research Topic collectively demonstrate how multimodal, neuroimaging, and computational approaches can advance our understanding of learning and cognitive states. They span methods from bibliometric analysis and neurophysiological measurement to deep learning and predictive modeling, showing how interdisciplinary perspectives can enhance assessment, diagnosis, and intervention in diverse learning and cognitive contexts.

The first article, *Research hotspots and trends of non-invasive vagus nerve stimulation: a bibliometric analysis from 2004 to 2023*, maps two decades of progress in non-invasive vagus nerve stimulation. By identifying global research trends, leading contributors, and emerging application areas, it provides a comprehensive overview that helps clarify how neuromodulation research informs clinical and cognitive domains (Chen et al.).

The second article, *Understanding emotional influences on sustained attention: a study using virtual reality and neurophysiological monitoring*, integrates VR-based emotion induction with EEG and PPG monitoring to explore how emotional valence and arousal affect sustained attention. Its findings highlight gender-specific patterns and demonstrate the value of immersive technologies and physiological data in studying emotion-attention interactions (Shen et al.).

The third article, *Automatic screening for posttraumatic stress disorder in early adolescents following the Ya'an earthquake using text mining techniques*, applies language models and machine-learning classifiers to self-narratives for early PTSD detection. This study exemplifies how text mining can transform qualitative self-reports into quantitative indicators of psychological states, improving early screening and intervention (Yuan et al.).

The fourth article, *Modulation of vigilance/alertness using beta (30 Hz) transcranial alternating current stimulation*, investigates how different stimulation parameters influence vigilance through behavioral performance measures. The results support the oscillatory nature of attentional vigilance and lay a foundation for closed-loop brain-stimulation interventions (Chu et al.).

The fifth article, *Development and validation of a postoperative delirium risk prediction model for non-cardiac surgery in elderly patients*, constructs and validates a predictive model for postoperative delirium by integrating cognitive assessments, sleep quality, and physiological indicators. It demonstrates how data-driven models can aid early clinical decision-making in cognitive risk management (Lin et al.).

The sixth article, *The application of radiomics in the diagnosis and evaluation of cognitive impairment related to neurological diseases*, reviews radiomic approaches for Alzheimer's disease, Parkinson's disease, and other neurological disorders. By summarizing imaging markers and analytical workflows, it underscores radiomics as an emerging multimodal method for early cognitive-impairment assessment (Xiao et al.).

The seventh article, *Advances in two-photon imaging for monitoring neural activity in behaving mice*, synthesizes recent progress in two-photon imaging and its applications in behaviorally engaged animal models. It highlights how fine-grained optical imaging links neural dynamics with behavioral outputs, expanding methodological toolkits for cognitive neuroscience (Li et al.).

The eighth article, *Cognitive training gain transfer in cognitively healthy aging: per protocol results of the German AgeGain study*, examines cognitive-training transfer effects and their neurobiological modulators using diffusion and functional MRI. It advances understanding of structural-functional connectivity mechanisms that support learning plasticity in healthy aging (Fischer et al.).

The ninth article, *Convolutional neural networks decode finger movements in motor sequence learning from MEG data*, validates a compact deep-learning model for decoding finger movements from non-invasive MEG signals. This work bridges machine learning and motor-learning neuroscience, illustrating efficient and interpretable decoding of fine-grained neural activity patterns (Zabolotniy et al.).

Finally, the tenth article, *Graph neural networks in Alzheimer's disease diagnosis: a review of unimodal and multimodal advances*, reviews the use of GNNs in processing multimodal neuroimaging data for Alzheimer's disease diagnosis. It systematically compares architectures, datasets, and performance metrics, offering future directions for integrating AI and neuroimaging in clinical cognitive assessment (Ali et al.).

Taken together, these studies illustrate the diversity and potential of multimodal approaches in cognitive neuroscience, ranging from non-invasive stimulation and imaging to AI-driven analytics, and offering methodological insights and translational implications for advancing learning and cognitive-state assessment.
